# The Effect of Multiple Firings on the Shear Bond Strength of Porcelain to a New Millable Alloy and a Conventional Casting Alloy

**DOI:** 10.3390/ma11040478

**Published:** 2018-03-22

**Authors:** Mitra Farzin, Rashin Giti, Amin Asalforush-Rezaiye

**Affiliations:** 1 Department of Prosthodontics, School of Dentistry, Shiraz University of Medical Sciences, Shiraz 7134814336, Iran; farzinm@sums.ac.ir; 2 Student Research Committee, School of Dentistry, Shiraz University of Medical Sciences, Shiraz 7134814336, Iran; aminasalforush@yahoo.com

**Keywords:** multiple firing, porcelain, bond strength, alloys

## Abstract

This study compared the effect of multiple firings on the shear bond strength (SBS) of porcelain to the new millable alloy (Ceramill Sintron) and a conventional casting alloy (4-all). Thirty-six cylindrical cores (6.8 × 9 mm) were made of millable and castable alloy through CAD/CAM and casting techniques, respectively (*n* = 18). In the center of each bar, a 4 × 4 × 2-mm shot of porcelain was fused. Having divided each group into 3 subgroups based on the number of firing cycles (3, 5, 7), the specimens were fixed in a universal testing machine and underwent a shear force test (1.5 mm/min crosshead speed) until fractured. Then the SBS values (MPa) were calculated, and the failure patterns were microscopically characterized as adhesive, cohesive, or mixed. Two-way ANOVA statistical test revealed that the number of porcelain firings had no significant effect on the SBS of any of the metal groups (*p* = 0.1); however, it was statistically higher in the millable group than the castable group (*p *< 0.05). Moreover, detecting the mixed failure pattern in all the specimens implied that the multiple firings had no significant effect on the failure pattern. The multiple porcelain firings had no significant effect on the SBS of porcelain to neither the millable nor castable alloys.

## 1. Introduction

Dental casting alloys play a prominent role in fabrication of porcelain fused to metal (PFM) restorations. In the 1980s, gold base alloys began to be replaced with base metal alloys in fixed dental prosthesis for their superior mechanical properties and their lower cost [1]. Compared with gold-base alloys, base metal alloys are stronger and harder, more resistant to distortion during porcelain firing, and have higher fusion temperature [2,3,4]. Meanwhile, they have overoxidation, uncertain biocompatibility, and finishing and polishing problems as their disadvantages [4]. 

Nickel-chromium (Ni-Cr) and cobalt-chromium (Co-Cr) alloys are typically used in base metal ceramic restoration [5]. In fabrication of PFM restorations, Ni-Cr alloys are preferred over gold-base alloys since they are harder, more elastic, and have higher fusion temperatures [5]. However, there are some disadvantages such as hypersensitivity to nickel caused by the release of Ni in the oral environment [6,7,8,9], which restricts the use of this alloy in 10–20% of the population [7]. Co-Cr alloys are used as the main structure of partial removable dental prostheses. They have fewer potential side effects and are more resistant to corrosion compared with Ni-Cr alloys [10]. In fixed prostheses, Co-Cr alloys are alternatively used for patients who are allergic to Ni [11]. 

The computer-aided design (CAD)/computer-aided manufacturing (CAM) technique has provided the chance of fabricating consistent and predictable dental restorations which has subsequently improved the quality of dental laboratory products [12,13,14,15]. A study reported better passive fit and the lowest stress around the implants whose frameworks were fabricated with CAD/CAM technique compared with the conventional ones [16]. Recent studies showed improved accuracy in the marginal and internal fitness of CAD/CAM fabricated metal-ceramic restorations, compared with the conventional ones [17,18]. 

The durability of metal-ceramic restorations relies on the bonding ability of porcelain veneer to the metal core [19]. The bond strength of these restorations is associated with several factors. Matching of the coefficient of thermal expansion (CTE) and contraction between the materials is among the determining factors for the metal-ceramic restorations to resist the stresses during heating and cooling cycles [20,21,22]. Chemical bonding is the primary mechanism of interaction between metal and ceramic [23,24], that is directly attributed to the composition and thickness of the surface oxide layer [25,26,27,28,29,30,31,32,33]. The absent or thin oxide layer would be thoroughly removed through ceramic sintering, and consequently reducing the bond strength. However, excessively thick oxide layer has poor cohesive strength [21]. 

On the other hand, to meet the esthetic and clinical requirements, the metal ceramic crowns should be subjected to an inevitable set of porcelain firings [34]. The firing cycles are likely to influence the oxide layer, and subsequently, the porcelain bond strength. Several studies showed that repeated firing of porcelain negatively affected the porcelain-metal compatibility and the bond strength [35,36,37]. 

In the early stages of CAD/CAM development, the Co-Cr was restrictedly used in PFM restorations because of the hardness of the alloy blocks [38]. The field of dentistry benefited from a limited number of CAD/CAM systems which could process the materials milled in final densely-sintered stage. Among the main problems of CAD/CAM systems are their high acquisition and maintenance costs. Besides the casting process, Co-Cr frameworks can also be fabricated by additive or subtractive processes through selective laser melting [37] and mechanical milling [39], respectively. 

Ceramill Sintron (AmannGirrbach; Koblach, Austria) is a recently-introduced presintered Co-Cr material which can be processed on desktop milling machines in-house. The manufacturing time and costs have been notably decreased in this product. Studies have reported that not only the bond strength of metal-ceramic combinations fabricated by these new techniques is comparable to those of casting, but also in most cases exhibit greater bond strengths [27,31,40,41,42]. 

Given the lack of relevant data, this study aimed to evaluate the shear bond strength (SBS) of porcelain to Ceramill Sintron new millable Co-Cr alloy after 3, 5 and 7 times of porcelain firing cycles and to compare it with a conventional Ni-Cr casting alloy (4-all; Ivoclar Vivadent; Schaan, Liechtenstein). The first null hypothesis was that multiple firings of porcelain would not decrease the metal-ceramic bond strength, and the second one was that the bond strength would not be different between the two alloys.

## 2. Materials and Methods 

This experimental study was performed on 36 cylindrical cores in two groups of Ceramill Sintron (*n* = 18) as a millable presintered Co-Cr alloy and 4-all (*n* = 18) as a castable base metal alloy to be subjected to various numbers of firing cycles. The cores were all 6.8 × 9 mm (diameter × height) and prepared by a dental technician according to the manufacturer’s instruction. Table 1 lists the materials used in this study.

Ceramill Sintron cores were designed by using CAD software (Remote DENTAL 2.0; imes-icore GmbH, Hamburg, Germany). To be processed, the presintered blanks were dry milled with milling machine (iMES-iCORECORiTEC 340i; Hamburg, Germany). Then, the cores were sintered under argon atmosphere at 1300°C in furnace (Magma preheating furnace; Renfert GmbH Hamburg, Germany) according to the manufacturer’s instructions.

To prepare 4-all cylindrical cores, CAD/CAM wax (Ceramill mall; Amann Girrbach; Koblach, Austria) was placed into the CAM milling machine to fabricate the wax cylinders. Then, the cores were cast by a casting machine (BEGO; Nautilus CC Plus; Lincoln, NE, USA). Finally, they were placed in a porcelain furnace (Programat P700/G2; Ivoclar Vivadent; Schaan, Liechtenstein) to form the oxidized layer.

The adhesive surface of cores was airborne-particle abraded with 110 µm Al_2_O_3_ particles (Basic Classic; Renfert GmbH) under 0.2 MPa gas pressure for 10 s at a standardized distance of 20 mm. They were finally put in ultrasonic bath of 95% methyl alcohol for 15 min, and then air dried.

**Figure 1:**
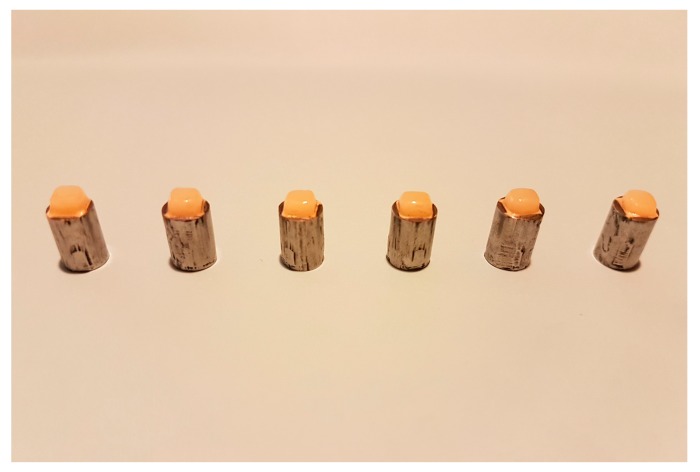
Metal cylinders with porcelain fused to their cross-section.

The first 3 porcelain firing cycles consisted of an initial wash of opaque porcelain, a layer of opaque porcelain, and a layer of dentin porcelain. First, a thin layer of wash opaque porcelain was applied to the central area (4 × 4 mm) of the metal core. Then, a layer of opaque porcelain and a layer of dentine porcelain were fired onto the previous layer by using the porcelain furnace. Dentine porcelain was built up as follows: the powder and liquid of the porcelain were mixed on a glass according to the manufacturer instruction and a quantity of the mixture was placed on the upper surface of core material. The porcelain mass was cut to the final dimensions of 4 × 4 × 2 mm with a No. 11 surgical blade and by using a digital caliper with accuracy up to 0.1 mm (Mini Electronic Caliper; Zhejiang, China). A 90 degrees cut was made vertically to the core. The dentine porcelain was then fired according to the manufacturer instruction [34]. The thickness of the total porcelain veneer was considered to be 2 mm (Figure 1). Finally, each metal group was divided into 3 subgroups (*n* = 6) to be subjected to various sets of firing cycles of 3, 5, and 7.

Silicone putty (Speedex putty; Altstatten, Switzerland) was used to make a female pattern of 20 × 8 × 30 mm (internal diameter × height × length) that would fix the specimen in the mounting mold. The specimen was inserted in one third of the putty pattern with a dental surveyor (Marathon-103; Saeyang; Daegu, Korea). Autopolymerizing resin (Acropars Re; Marlic; Tehran, Iran) was poured into the pattern. A surveyor was used to embed the specimen in a way that only 1 mm of the metal was exposed above the resin surface.

**Figure 2:**
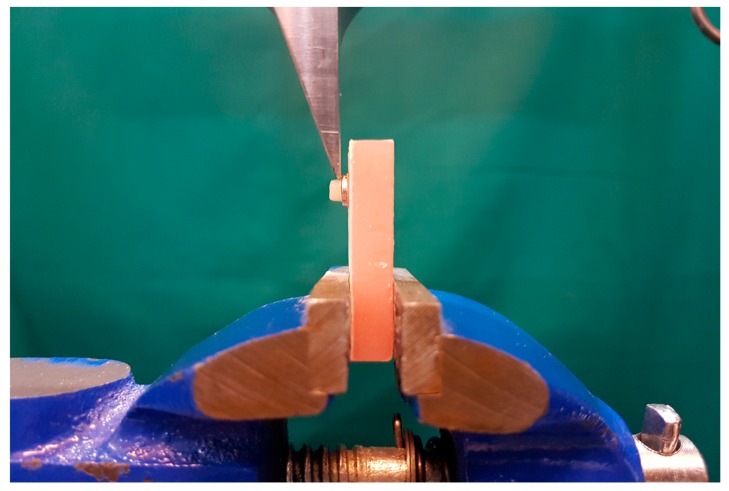
Shear bond strength testing.

To measure the adherence of specimens according to ISO standard 9693, the porcelain fracture was tested by using a universal testing machine (Zwick/Roell Z020; Zwick GmbH; Kennesaw, CA, USA). The specimens embedded in resin molds were put inside a shear test jig, which was then fixed in a universal testing machine (Instron Inc., Norwood, MA, USA) (Figure 2). To focus the shear loading on the interface of the core and veneer, it was tried to aim the tip of the Instron mono-bevel blade at the place where the two parts met. The shear force was applied at a constant crosshead speed of 1.5 mm/min until failure occurred. The maximum force leading to failure was recorded, and the fractured area was measured with a Vernier caliper. To calculate the average shear bond strength (MPa), the maximum failure force (N) was divided by the cross-sectional bonding area of each specimen (mm^2^).

The failure patterns were analyzed by inspecting the fractured surfaces under a scanning electron microscopy (SEM) (Cambridge S-360, CAMBRIDGE, Austin, Texas, USA) at ×30 and ×1000 magnifications. The failure modes were classified into 3 types of adhesive (complete delamination of veneering porcelain from the metal), cohesive (internal fracture of the veneering porcelain or core metal), and mixed failure (combination of cohesive and adhesive failure within the same bond).

Statistical analysis was performed using SPSS software (SPSS Inc.; version 20, Chicago, IL, USA). Kolmogorove-Smirnov test was performed to assess the hypothesis of normal distribution. The assumption of homogeneity of variances among groups was tested using Levene’s F test. The mean SBS of each group were analyzed by two-way ANOVA, with the SBS as the dependent variable and the alloys and porcelain firing cycles as the independent factors. *p* values < 0.05 were statistically significant in all tests.

## 3. Results

**Figure 3:**
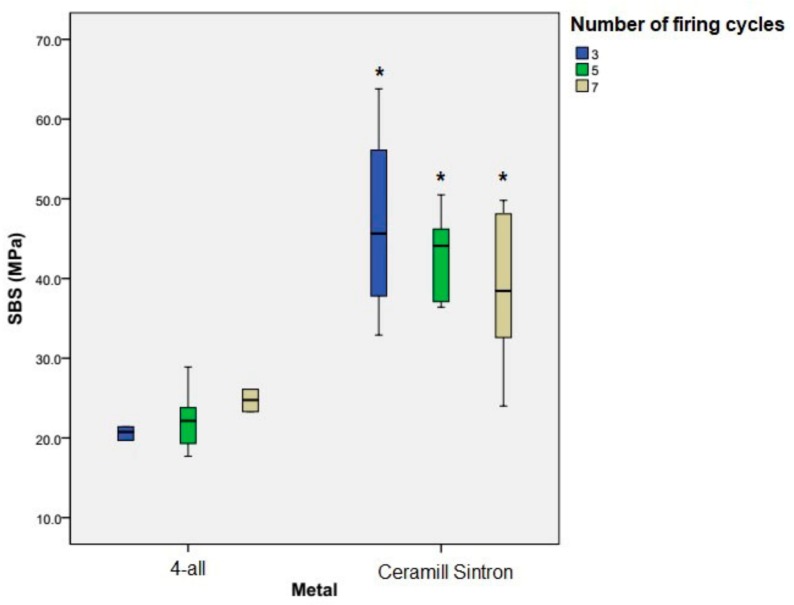
The mean and standard deviation of the shear bond strength (MPa). The shear bond strength values of Ceramill Sintron group in all numbers of firing cycles were significantly higher than the 4-all group (*: *p*-value < 0.05).

The means and standard deviations of the shear bond strength of both metal groups are presented in Table 2 and Figure 3, based on the number of firing cycles. Kolmogorov-Smirnov and Levene’s tests revealed that data were normally distributed, and the variances were homogenous. Besides, Table 3 summarizes the results oftwo-way ANOVA. According to the findings of this study, the number of porcelain firing cycles had no significant effect on the SBS of any of the metal groups (*p* = 0.7). However, as the number of porcelain firing cycles increased from 3 to 7, Ceramill Sintron showed decrease in the SBS. Neither was any relation noted between the type of metal and number of firing cycles (*p *= 0.1). Meanwhile, the mean bond strength in Ceramill Sintron group (42.872) was significantly (*p *< 0.001) much greater than that in 4-all group (22.489).

**Figure 4:**
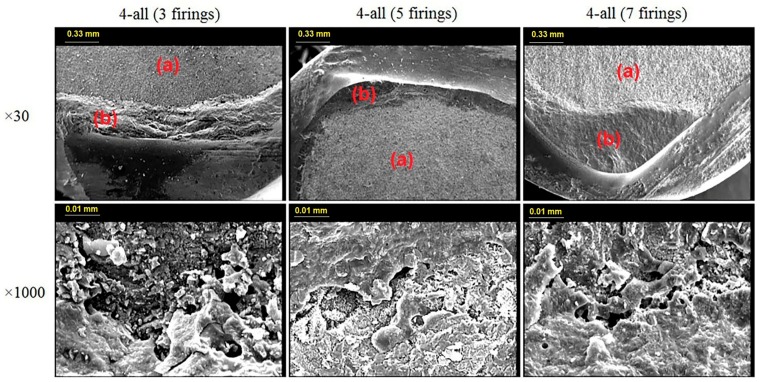
×30 and ×1000 magnification of mixed failure pattern of 4-all group after 3, 5 and 7 firings. (a) is the adhesive and (b) is the cohesive part of mixed failure patterns.

**Figure 5:**
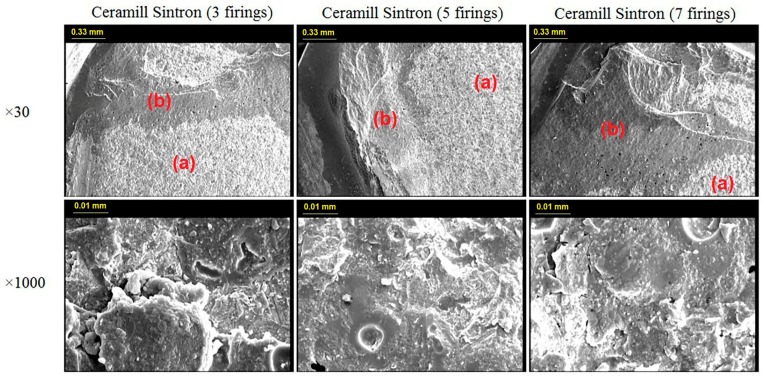
×30 and ×1000 magnification of mixed failure pattern of Ceramill Sintron group after 3, 5 and 7 firings. (a) is the adhesive and (b) is the cohesive part of mixed failure patterns.

Figure 4 and Figure 5 display the representative SEM photomicrographs (×30 and ×1000 magnifications) from the fractured surfaces of the two metal groups after 3, 5, and 7 firing cycles. In neither the CAD/CAM nor the casting group, the fracture mode was affected by the number of firing cycles and the type of metals. All the specimens had mixed failure pattern at the debonding areas; that is, a mixture of adhesive fracture mode between the alloy and the oxide, as well as cohesive fracture mode within the porcelain were observed. However, the porcelain adherence surfaces on the debonding areas were obviously larger in the CAD/CAM metal group than the cast metal group.

## 4. Discussion

The present in vitro study investigated the metal-ceramic SBS and failure modes of a castable Ni-Cr alloy (4-all) and a new presintered millable Co-Cr alloy (Ceramill Sintron) after 3, 5, and 7 porcelain firing cycles. Based on the findings, the first null hypothesis could be accepted since increased number of firing cycles had no significant effect on the SBS of the two metal groups. 

Different authors surveyed the effect of multiple firings on bond strength of porcelain to different alloys [34,43,44,45]. Ren et al. showed that increasing the number of porcelain firing from 3 to 5 and 7 cycles had no significant effect on the bond strength of porcelain to two different base metal alloys (selective laser melting and conventional casting Co-Cr alloy) used in their study [34]. Likewise, Stannard et al. detected that porcelain multiple firing had no statistically significant effect on the porcelain bond strength to a noble metal [45]. In a study by Barghi et al., no significant change was observed in the bond strength of a base metal alloy by increasing the porcelain firing cycles [43]. 

The results of the present study also confirmed the inadequacy of multiple firing on the SBS of porcelain to any of the studied alloys. Seemingly, repeated firings under controlled conditions did not significantly change the bond strength of the two apparently well-matched porcelain-metal alloy combinations.

The second null hypothesis could also be rejected due to the significant differences of the SBS between the two metal groups after multiple firings. According to the results, the mean bond strength of the new millable alloy (42.87 MPa) was significantly higher than the bond strength of the cast alloy (22.48 MPa), which could play a prominent role in long-term survival of the metal-ceramic restorations against the shear stresses. 

Stawarczyk et al. in 2014 found that the metal-ceramic bond strength of the millable alloy (Ceramill Sintron) was statistically comparable to those of cast (Girobond NB) and laser-sintered (Ceramill NP) alloys [31]. Wang et al. in 2016 evaluated the metal-ceramic bond strength of three dental Co-Cr alloys fabricated by casting, milling, and selective laser melting (SLM) techniques. They noted that Co-Cr alloy, fabricated through the SLM and milling techniques, had stronger metal-ceramic bond than the cast alloy [33]. 

The lower bond strength of the cast group in both studies was attributed to the increased thickness of the oxide layer on metal-veneer interface. Both studies reported the highest oxide surface layers in the cast group and the lowest in milling group. It seems that the process of casting and melting of the alloy might increase the thickness of the surface oxide layer and reduce the bond strength of the metal-porcelain combination [27,31]. 

The diffusion of the alloy surface oxides and the ceramic results in a chemisorption which creates the metal-ceramic bond. In fact, when the alloy gets wet with the veneering ceramic and the veneering ceramic is then fired, these oxides are formed. Several studies showed that the thicker the oxide layer was, the lower the bond strength of metal-ceramic in base metal alloys would be [27,28,29,30,31,32]. 

McLean and Sced announced that the quality of metal-ceramic bond strength was highly determined by the constituents of the surface oxide layer. They observed that the surface oxide of Ni-Cr alloy under high temperature was mainly made of NiO and Cr_2_O_3_ which is the most abundant type of oxide product. A relationship was noted between the amount of Cr_2_O_3_ in oxide layer and the Cr weight percent (wt %) in the alloy. They stated that the excessive Cr_2_O_3_ could change the thermal expansion coefficient of the porcelain layer, and thus, induce internal stress, which in turn reduce the bonding strength between the metal and porcelain [46]. 

On the other hand, presence of the aluminum (Al) element in alloy composition improves the degree of oxidation through formation of Al_2_O_3_, which consequently reduces the thickness of the oxide layer [47,48]. The composition of both alloys used in this study contained approximately the same level of Cr (25.7 wt % in 4-all and 28 wt % in Ceramill Sintron), and almost no Al, which made them susceptible to metal-ceramic bond failure through the oxide layer. 

Beside the Cr and Al elements, molybdenum (Mo) is the third most important element in these alloys. Mo is a refractory metal and not readily oxidized, but the oxide product of Mo (e.g., MoO_3_) becomes volatile at temperatures exceeding 700°C [47]. In the above-mentioned study [46], the alloys containing more than 5 wt % of Mo were found to have a relatively higher amount of MoO_3_ volatized during heat treatment and leaving the surface oxide layer with more voids and/or defects. These all led to a lower metal-ceramic bond strength. On the other hand, since Mo decreases the coefficient of expansion [48], presence of Mo (≤5wt %) in the alloy seems to minimize the appearance of the voids in the surface oxide structure, besides neutralizing the changes induced by Cr_2_O_3_ in the thermal expansion coefficient.

Due to the relative equivalence of Cr in the combination of Ni-Cr and Co-Cr alloys, the Mo percentage in each alloy can highly influence the properties of their surface oxide layers. In the present study, Ceramill Sintron contained 5 wt % of Mo in its composition, which was expected to increase the consistency of the CTE of the alloy and porcelain. Moreover, according to McLean and Sced’s study [46], this amount of Mo might decrease the formation of plausible voids in the surface oxide layer. In contrast, 4-all contained 11 wt % of Mo, which can be considered as a reason of the low metal-ceramic bond strength of this alloy compared with Ceramill Sintron.

The metal-ceramic bonding ability is not only influenced by the surface oxide layer, but also the matching of CTE between metal and the veneering porcelain. The difference of CTE between the porcelain and metal, being slightly lower in the former, results in a compression of porcelain when cooling from firing temperatures and facilitates the metal-ceramic bond [20]. However, when the difference of CTE between the two materials is higher than 1 × 10^–6^/degree Celsius (°C), their contraction occurs at quite different rates, which consequently increases the residual stresses across the interface and causes delamination of the porcelain from the metal substrate [20,22]. 

The selection of materials in this study was based on optimal CTE compatibility between the alloys and the porcelain. The CTE of alloys used in this study was between 14.1–14.5 × 10^–6^/degree Celsius (°C). It was within the range determined by the veneering porcelain manufacturer (13.5–15.0 × 10^–6^/degree Celsius (°C)). Therefore, the impact of this factor on the result was minimized. On the other hand, the improved behavior of the milling alloy might be explained by the more regular microstructural arrangement of the milling alloys [34]. 

In addition to the shear bond test, this study also investigated the metal-ceramic bond strength through SEM analyses as an additional evaluation. The observations revealed a combination of cohesive and adhesive fracture modes (mixed fracture) in all the specimens. Based on microscopic analyses, more metal exposure was detected on the cast Ni-Cr metal-ceramic interface. 

Ren et al. did not find any significant difference between the metal-ceramic bond strength of SLM Co-Cr alloy and the conventionally cast Co-Cr groups [34]. However, the microscopic analyses revealed more metal exposure on the metal-ceramic interface of the cast Co-Cr than the SLM specimens. It indicated that the SLM group had a stronger bond with the porcelain veneers. Likewise, Li et al. conducted a microscopic analysis of the metal-ceramic interface of three different Co-Cr alloys (casting, milling, and selective laser melting) [49]. They detected significantly higher porcelain adherence in the milled and SLM groups than the cast group. Both studies attributed the improved behavior to the microstructure changes of the alloys caused through different manufacturing methods. Scanning electron microscopy revealed a more regular arrangement in the CAD/CAM specimens, which might justify their stronger chemical bond with porcelain [34,49]. 

Base on the American National Standards Institute/American Dental Association specification 38 (2000) and ISO standard 9693:2012 for evaluation of metal-ceramic bond, the minimum acceptable bond strength is set at 25 MPa [50]. In the current study, the mean bond strength values for all the specimens of Ceramill Sintron alloy exceeded the minimum value, indicating that the metal-ceramic bond strength of samples fabricated through milling technique lies within a clinically acceptable range. The results of this in vitro study also showed that the mean bond strength values of 4-all casting groups in all numbers of firing cycles were below the minimum acceptable bond strength. It implies that the computerized CAD/CAM technique can help enhancing the bond strength at least to the clinically minimum acceptable threshold.

The pre-sintered CAD/CAM blanks allow manufacturing in small laboratory CAD/CAM systems as the material is easily machinable. This might be an attractive alternative to dental laboratories with CAD/CAM technology to mill the base metal alloys instead of casting. Furthermore, manufacturing the metal-ceramic restorations with this new alloy is free of many of the disadvantages of the traditional methods such as time-consumingness, human skills dependence, multiple and complex stages, and difficult finishing. Additionally, the absence of nickel in this new alloy eliminates the plausible allergic reactions to this element in susceptible patients.

Among the several limitations of this study was the unavailability of measuring the Si content of specimens with scanning electron microscopy (SEM) and energy-dispersive X-ray spectroscopy (EDS) to investigate the chemical structure of the surface oxide layer in the alloys, which could have provided useful information on the effect of multiple firing on the composition of this layer and its bond strength. Besides, this research investigated only one commercial brand of CAD/CAM millable alloys. Further studies are suggested to survey the effect of multiple firings on the shear bond strength of metal-ceramic restorations made of other CAD/CAM millable Co-Cr alloys.

## 5. Conclusions

Within the limitations of this study, it can be concluded that multiple porcelain firing cycles has no significant effect on the shear bond strength of porcelain to both casting and millable alloys (*p* = 0.7). It was also noted that in all the 3, 5, and 7 firing cycles, the millable group exhibited superior metal-ceramic bond strength that exceeded the requirements of ISO standard 9693:2012 (*p *< 0.001). Moreover, the bonding surface of the millable alloy showed better behavior than the conventional casting alloy. The conclusion can also be drawn that the millable Co-Cr alloy can be used as an alternative to the casting alloy for metal-ceramic restorations and CAD/CAM manufacturing can be efficiently used in PFM restorations.

## Figures and Tables

**Table 1:** Brand, manufacturer, and composition of the employed materials.

Materials	Brand	Manufacturer	Composition (wt %)
Core	Ceramill Sintron	AmannGirrbach	Co 66, Cr 28, Mo 5, Si < 1, Fe < 1, Mn < 1
	4-all	Ivoclar Vivadent	Ni 61.4, Cr 25.7, Mo 11.0, Si 1.5, Mn < 1.0, Al < 1.0, C < 1.0
Porcelain	IPS InLine SystemOpaquer	Ivoclar Vivadent	
	IPS InLine Dentin	Ivoclar Vivadent	

**Table 2:** Mean (standard deviation) shear bond strength of the two metal groups according to the firing porcelain cycles.

Metal Groups	Mean and Std. Deviation of SBS (MPa)			
	3 Firing Cycles	5 Firing Cycles	7 Firing Cycles	Total
4-all	20.41 ± 2.65	22.33 ± 3.94	24.71 ± 4.29	22.48 ±3.91
Ceramill Sintron	46.98 ± 11.43	43.06 ± 5.52	38.56 ± 9.66	42.87 ± 9.34

**Table 3:** Results of the 2-way ANOVA.

Source	Sum of Squares	df	Mean Square	F	*p*
A. Metal type	3739.322	1	3739.322	75.905	<0.001
B. Number of firing	25.427	2	12.714	0.258	0.774
Interactions A and B	243.122	2	121.561	2.468	0.102
Error	1477.905	30	49.264	-	-
Total	5485.776	35	-	-	-
